# Interspecies transfer of vancomycin, erythromycin and tetracycline resistance among *Enterococcus* species recovered from agrarian sources

**DOI:** 10.1186/s12866-017-0928-3

**Published:** 2017-01-18

**Authors:** M. Conwell, V. Daniels, P. J. Naughton, J. S. G. Dooley

**Affiliations:** School of Biomedical Sciences, Ulster University, Cromore Road, Coleraine, BT52 1SA UK

**Keywords:** Enterococci, Water, Conjugation, Vancomycin resistance, Erythromycin resistance, Tetracycline resistance

## Abstract

**Background:**

Enterococci are now well recognised for their ability to transfer antibiotic resistance and for their association with nosocomial infections, but less is known regarding their relevance in the wider environment. *Enterococcus faecalis* and *Enterococcus faecium* were isolated from a range of agrarian associated sources (low-flow water, septic tank, poultry litter, high flow water, slurry/soil) and were assessed for latent ability to transfer antimicrobial resistance.

**Results:**

The isolates were tested for phenotypic clumping in the presence of cell-free supernatant from other isolates. Some isolates were identified which demonstrated clumping, indicating that they possessed peptide sex pheromone conjugal machinery. All isolates were also tested for antibiotic resistance phenotypes using both disc diffusion and minimum inhibitory concentration (MIC) assays. These tests revealed that the enterococci demonstrated both phenotypic clumping and antibiotic resistance phenotypes. Based on these selection criteria, the isolates were identified as having the potential for horizontal gene transfer and were used to investigate the transfer of multiple antibiotic resistance phenotypes. Conjugal transfer of antibiotic resistance phenotypes was determined using a solid agar mating method followed by a standard antibiotic selection test resulting in different transfer patterns. An interspecies conjugal transfer of vancomycin resistance from *E. faecalis* to *E. faecium* was identified while the remaining reactions were within the same species. Transfer efficiencies ranging from 2 × 10^−1^ to 2.3 × 10^−5^ were determined based on the reactions of three donor isolates (MF06036, MF0410 and MF06035) and two recipient isolates (MW01105^Rif^ and ST01109^Rif^), with the transfer of vancomycin, erythromycin and tetracycline resistance genes.

**Conclusions:**

The conjugation reactions and selection conditions used in this study resulted in a variety of co-transferred resistance phenotypes suggesting the presence of different mobile elements in the set of natural isolates. This study highlights the potential for extensive horizontal gene transfer in a previously neglected reservoir for enterococci*.*

## Background

Enterococci are Gram positive, facultative anaerobes and despite not forming spores they are tolerant to a range of environmental conditions [1]. They are ubiquitous inhabitants of the mammalian gastrointestinal tract and are regularly recovered from agrarian ecosystems [2, 3]. Although they do not thrive in water they have been found in surface waters prone to faecal contamination [4] and because of their resistance to salinity they also pose a threat to recreational bathing waters [5, 6]. Farming practices in Ireland and the United Kingdom currently rely on significant amounts of therapeutic antibiotics and much of the farming effluent drains directly into local water ecosystems [7, 8]. Recent decades have seen an inexorable rise in antibiotic resistance within enterococci with the emergence of multi-resistance being of particular concern [9]. Hence, water catchments close to farmland have the potential to provide a reservoir of antibiotic resistant enterococci which may pose a threat to human health.

Among the enterococci, a conjugation system based upon pheromone signalling [10] has been implicated in the spread of some of the most important resistance phenotypes although its’ influence under environmental conditions remains to be determined. Plasmids that respond to pheromone are common in *E. faecalis* but less common in *E. faecium* [9]. These plasmids have also been shown to carry a number of virulence traits including: bacteriocin and cytolysin production, and promote donor-recipient hybrid genomes encoding, for example vancomycin and tetracycline resistance [11]. Enterococci are increasingly presenting as nosocomial pathogens [12] and antibiotic resistance has now become a permanent feature in human infectious diseases. These, pheromone based conjugation systems may also be responsible for the interspecies spread of antibiotic resistance genes amongst staphylococci and enterococci [13]. Previous studies have investigated the presence of antibiotic resistant enterococci in the environment, humans and foodstuffs but this is the first study to focus on enterococci isolated from sites in an agrarian ecosystem.

In the current study enterococci isolated from an agrarian ecosystem were examined to assess their resistance phenotypes and ability to transfer resistance genes by conjugation. These processes identify the catchments as potential reservoirs of antibiotic gene transfer in enterococci. This study set out to demonstrate conjugal transfer of a range of antibiotic resistance genes from enterococci, with multi-resistant strains exhibiting a high level of horizontal gene transfer ability. The aim of this study was to examine the presence of antibiotic resistance genes in water-borne enterococci and determine the phenotypes and genotypes of these isolates with a view towards investigating the transferability of antimicrobial resistance determinants between different species of enterococci.

## Methods

### Bacterial strains and reagents

The *E. faecalis* and *E. faecium* used in this study (Table 1), were previously isolated from various sources feeding into river headwaters [14]. Briefly, water was collected and filtered using Millipore Microfil membrane filtration system with 0.45 μm filters (Millipore. Hertfordshire, UK). Volumes of 1-50 ml of water samples (diluted in maximum recovery diluent) were filtered and grown on Slanetz and Bartley agar (Oxoid CM0337). Plates were incubated for 4 h at 37 °C and 44 h at 42 °C. Phenotypic identification of isolates as enterococci was carried out using: aesculin hydrolysis, PhenePlate™ analysis, gram staining, catalase, PYRase and azide tests [14–16]. All bacteria were maintained on tryptone soy agar (TSA), (Oxoid CM0131) at 4 °C throughout the experimental time period. All growth conditions and experiments, unless specifically mentioned, were carried out on tryptone soya broth (TSB), (Oxoid CM0129) or TSA at 37 °C. All chemicals and antibiotics used were obtained from Sigma, unless otherwise stated.

**Table 1:** Enterococci (species, source and role in conjugation) in solid surface mating experiments

Name and Species of agrarian enterococci			
Isolate	Species	Source	Potential role^a^
MF06035	*E. Faecalis*	Poultry litter	Donor
MF06036	*E. Faecalis*	Poultry litter	Donor
MW01038	*E. Faecalis*	Low-flow water	Donor
MW02102	*E. Faecalis*	Low-flow water	Donor
MW03020	*E. Faecalis*	Low-flow water	Donor
MW03025	*E. Faecalis*	Low-flow water	Donor
MW03051	*E. Faecalis*	Low-flow water	Donor
ST02011	*E. Faecalis*	High-flow water	Donor
MF06019	*E. Faecium*	Poultry litter	Donor
MF04010	*E. Faecalis*	Slurry/soil	Donor
MF06030	*E. Faecium*	Poultry litter	Donor
MW03061	*E. Faecium*	low flow water	Donor
MW01105	*E. Faecalis*	Low-flow water	Recipient
MW02043	*E. Faecalis*	Low-flow water	Recipient
ST01109	*E. Faecium*	Septic tank	Recipient

^a^Based on results of clumping assays

### Pheromone-induced clumping assay: identification of recipients and donors

To test if an isolate could be a pheromone producing recipient or a potential donor, cells were grown for 16 h in 20 ml of TSB. Cells were pelleted by centrifugation (10,000 RCF) for 15 min at 4 °C. Supernatant was removed and filter sterilised (Millipore 0.22 micron filters) providing a pheromone-enriched broth. The clumping assay consisted of 500 μl pheromone-enriched broth, 500 μl fresh TSB, and 20 μl of a 16 h culture added in a 1.5 ml Eppendorf and incubated for four hours (37 °C rotating at 150 rpm). From the final suspension, 20 μl was dropped on a glass slide and a coverslip (22 mm) was applied. Clumping was determined by eye. Isolates that induced clumping but did not clump in the presence of other supernatants were deemed potential recipients. Isolates that readily clumped were characterised as potential donors. Cells were imaged using phase contrast with 100x (Nikon plan fluor 1.3 oil ph3 DLL) on a Nikon eclipse E400 with a Nikon DS-fi1c. Images were captured with NIS-elements and imageJ (NIH).

### Antibiotic resistance testing

Enterococci were selected for testing based on positive results from the clumping assay either as a recipient or donor for conjugation. Prior to Minimum inhibitory concentration (MIC) testing, the Kirby-Bauer disc diffusion assay was performed on Mueller-Hinton agar (CM0405) to obtain an estimate of the antibiotic resistance phenotype for each isolate. Briefly, an 18 h culture of bacteria was re-suspended in phosphate buffered saline. The bacterial suspension was swabbed and spread over the surface of a dried plate. Antibiotic discs (Oxoid) were stamped on the plates using a disc dispenser (Oxoid ST6090). Antibiotics used were ampicillin (10 μg), amoxycillin (25 μg), cephalothin (30 μg), ciprofloxacin (5 μg), erythromycin (30 μg), gentamicin (120 μg), impenem (10 μg), linezolid (10 μg), neomycin (30 μg), oxytetracycline (30 μg), quinupristin/dalfopristin (15 μg), streptomycin (25 μg), trimethoprim (5 μg), tetracycline (30 μg), teicoplanin (30 μg), and vancomycin (30 μg). Plates were incubated for 24 h at 37 °C. Zones were measured (mm) and compared to EUCAST ECOFFs [17]. *E. faecalis* ATCC 29212 was used as a susceptibility control.

Minimum inhibitory concentration (MIC) assays were carried out on the donors that demonstrated positive antibiotic resistance in the disc diffusion assay. The protocol utilised the broth microdilution protocol described previously [18] with the addition of p-Iodonitrotetrazolium [19], as a colorimetric indicator. Based on the results of the Kirby-Bauer tests the antibiotics used were: vancomycin, erythromycin, streptomycin, tetracycline, trimethoprim, teicoplanin, rifampicin, kanamycin (Gibco), chloramphenicol, and gentamycin. All experiments were carried out using Muller Hinton broth (Oxoid, CM0405) and Iso-Sensitest broth (Oxoid, CM0471). Trimethoprim, resistance/susceptibility was determined in accordance with the EUCAST value database [17].

### Creation of rifampicin resistant recipients

In order to facilitate counter-selection in conjugation experiments, it was necessary to generate rifampicin resistance among the recipient strains. The rifampicin sensitive pheromone producing isolates were grown to late log phase in sub-minimum inhibitory concentrations of the antibiotic. Successive generations were sub-cultured in increasing concentrations of the antibiotic until isolates could grow in 512 mg/L of rifampicin. All donor isolates in this study were susceptible to rifampicin and all recipients were resistant to rifampicin (denoted as ^Rif^).

### Determination of transfer efficiency of resistance

Conjugation experiments were carried out using the solid agar mating method [20]. Briefly, donor and recipient isolates were added together at a ratio of 1:9 onto a non-selective TSA plate and allowed to conjugate for 24 h. The resulting lawn was re-suspended in 1 ml of PBS, serially diluted and spread onto TSA selection plates containing appropriate antibiotic combinations. Plates were incubated for a further 24 h and transfer efficiencies were calculated (number of transconjugants per donor). Selection plates were comprised of TSA with rifampicin (100 μg/ml) and either vancomycin (10 μg/ml); erythromycin (50 μg/ml); tetracycline (16 μg/ml); or kanamycin (512 μg/ml). Antibiotic free TSA was used as a control. All conjugation reactions were performed at 37 °C.

### Detection of antibiotic resistance genes in the transconjugants and their donors

Resistance genes were detected using colony PCR [21]. Two hundred ng template DNA (in 1 μl) was added to 19 μl of mastermix, containing final concentrations of 1.5 mM of Mg^2+^ (2 μl of 1x PCR buffer and 0.6 μl of separate MgCl_2_), 0.2 mM dNTP’s each, 0.5 μM forward and reverse primer and 1.0U of Taq polymerase. Primer sequences (Table 2) were selected based on antibiotic genes known from the literature to be associated with the MIC phenotypes of interest in this study. All PCR reactions were run for 30 cycles with a final extension of five minutes. Samples were analysed by electrophoresis with Tris-Borate EDTA (TBE), in 1.5% agarose with ethidium bromide (final concentration 0.5 μg/ml) at 100 volts and visualised on an Alpha Imager (Cell biosciences Heidelberg, Germany).

**Table 2:** Primer sequences for antibiotic resistance genes

List of primers used				
Gene	Primer	Sequence 5′ to 3′	Product Size	References
tetK	tetK F	TTAGGTGAAGGGTTAGGTCC	718	[31]
	tetK R	GCAAACTCATTCCAGAAGCA		
tetL	tetL F	ATAAATTGTTTCGGGTCGGTAAT	1077	[32]
	tetL R	AACCAGCCAACTAATGACAATGAT		
tetM	tetM F	GTTAAATAGTGTTCTTGGAG	657	[31]
	tetM R	CTAAGATATGGCTCTAACAA		
tetO	tetO F	GATGGCATACAGGCACAGAC	614	[33]
	tetO R	CAATATCACCAGAGCAGGCT		
tetS	tetS F	TGGAACGCCAGAGAGGTATT	660	[33]
	tetS R	ACATAGACAAGCCGTTGACC		
tetT	tetT F	AAGGTTTATTATATAAAAGTG	169	[34]
	tetT R	AGGTGTATCTATGATATTTAC		
tetW	tetW F	GAGAGCCTGCTATATGCCAGC	168	[33]
	tetW R	GGGCGTATCCACAATGTTAAC		
ErmB	ermB F	AGGGTTGCTCTTGCACACTC	119	This study^1^
	ermB R	CTGTGGTATGGCGGGTAAGT		
VanA	vanA F	CTACTCCCGCCTTTTGGGTT	109	This study^2^
	vanA R	TTCACACCGAAGGATGAGCC		

^1^: >gi|488766672:c26630-25893, ^2^:>gi|305678685:28460–29491

## Results

**Fig. 1:**
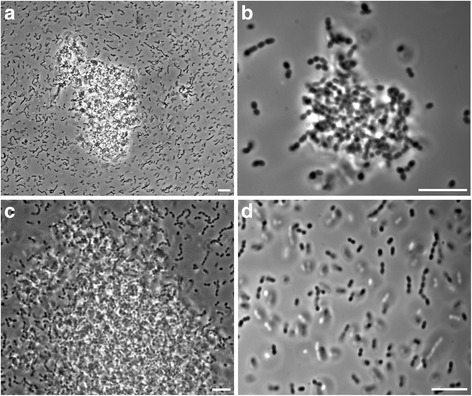
Phenotypic clumping of enterococci examined with phase contrast microscopy. All potential donor isolates of enterococci were subjected to exposure of supernatant from each of the three pheromone producers for four hours in a 24 well microplate at 37 °C. (A) 20x micrograph of *E. Faecalis* MF06036 with the supernatant of *E. Faecalis* MW01105^Rif^, (centre), the typical reaction observed when aggregation signalling is activated. (B) 100x micrograph of MF06036 with the supernatant from *E. Faecium* ST01109^Rif^. (C) 40x image of a large reaction between *E. Faecalis* MF06035 and the supernatant of MW01105^Rif^. (D) 100x Negative clumping reaction of MF06036 supernatant added to MW01105^Rif^. Scale bars represent ten microns

The enterococci recovered from a variety of agrarian sources (Table 1) were tested for antibiotic resistance phenotypes as well as their pheromone-induced clumping ability, to identify a subgroup of isolates that were likely to undergo horizontal gene transfer by conjugation. A clumping reaction manifested as a large collection of densely packed immobile cells surrounded by planktonic cells (Fig. 1). Positive clumping reactions occurred within the same species as well as between members of different species e.g. ST01109^Rif^ (*E. faecium*) cell-free supernatant induced clumping when added to MF06036 (*E. faecalis*) (Table 3). Eleven isolates of *E. faecalis* and four isolates of *E. faecium* were selected based on clumping ability and antimicrobial resistance phenotype as having potential to transfer antibiotic resistance by conjugation.

**Table 3:** Clumping of donor isolates by supernatant of recipient isolates

Recipients			
	MW01105	ST01109	MW02043
Donor Isolates			
MF06035	+	+	ND
MF06036	+	+	ND
MW01038	+	ND	+
MW02102	+	ND	ND
MW03020	+	+	+
MW03025	+	+	+
MW03051	+	ND	+
ST02011	ND	+	ND
MF06019	+	+	+
MF04010	+	+	+
MF06030	+	+	+
MW03061	ND	+	ND

Positive clumping phenotypes presented when supernatant was added to potential donor isolates (Lab identifier numbers). MW01105, MW02043 and ST01109 induce clumping and therefore do not react to supernatants. MW01105 induces clumping in 9 isolates; ST01109 induces clumping in 8 isolates; and ST01109 induces clumping in 7 isolates. Donor isolates such as MW03025 and MF04010 can be induced to clump by all three supernatants. Other Donors such as ST02011 can only be induced to clump by one supernatant (ST01109). *ND* none detected

### Clumping assay to identify potential donor and recipient enterococci

The prospective ability to conjugate was demonstrated by clumping of potential donor cells in the presence of cell free supernatant from a potential recipient (Fig. 1). Of the isolates screened MW01105, MW02043 and ST01109 did not have the ability to clump under these conditions and were therefore designated as potential recipients. The remaining isolates were considered potential donor isolates as they could be induced to clump. We observed a number of reactions including some strains that gave a highly- specific clumping profile and only reacted to supernatant from one other strain e.g. MW02102 and ST02011 exhibited a single clumping reaction while others demonstrated the potential for promiscuity by reacting to supernatants from a number of strains e.g. MW03025 and MF04010 readily reacted to the pheromone of all the recipient isolates.

### Antibiotic resistance profiles of donor and recipient enterococci

Of the isolates tested, most demonstrated at least two antibiotic resistance phenotypes (Table 4). MF06035 was highly resistant to five antibiotics and MF06036 was resistant to six antibiotics (Table 4). The isolates (MW01038-ST02011) appeared to show a typical susceptibility to vancomycin in broth microdilution assays. Additionally, ST02011 was highly resistant to kanamycin having an MIC of 2048 μg/ml. MF06019 was highly resistant to erythromycin (x256 higher compared to ECOFF values (Table 4)) and kanamycin. MF04010 was one of two isolates that was highly resistant to tetracycline (x32 higher than the ECOFF values). MF06030 was resistant to tetracycline and highly resistant to kanamycin, and MF03061 was also resistant to tetracycline. MW01105^Rif^ was highly resistant to streptomycin (at least four times more resistant than the other recipients) and is a highly resistant rifampicin mutant. MW02043^Rif^ was highly resistant to kanamycin and was also a rifampicin resistant mutant. ST01109^Rif^ was a highly resistant rifampicin mutant that was completely susceptible to all tested antibiotics.

**Table 4:** Antibiotic resistance profiles of enterococcus isolates and transconjugants

Enterococcal isolates and transconjugants	MIC (μg/ml) at 24 h						
	VA	E	SM	TET	TMP	TE	KAN
*E.faecalis* MF06035	R	R	R	R	R	R	64
*E.faecalis* MF06036	R	R	R	R	R	R	64
*E.faecalis* MW01038	4	<0.25	256	<0.5	R	0.5	256
*E.faecalis* MW02102	4	<0.25	256	<0.5	R	1	64
*E.faecalis* MW03020	4	<0.25	256	R	0.25	<0.25	64
*E.faecalis* MW03025	R	<0.25	256	1	1	<0.25	64
*E.faecalis* MW03051	4	<0.25	256	1	0.5	1	64
*E.faecalis* ST02011	4	32^R^	128	<0.5	0.25	<0.25	1024
*E.faecium* MF06019	1	R	64	<0.5	0.06	<0.25	>1024^HLR^
*E.faecalis* MF04010	1	<0.25	R	R	0.13	<0.25	256
*E.faecalis* MF06030	0.5	<0.25	64	R	0.13	<0.25	>1024^HLR^
*E.faecium* MF03061	1	1	32	R	0.06	<0.25	1024
*E.faecalis* MW01105^Rif^	1	0.5	R	<0.5	0.13	2	128
*E.faecalis* MW02043^Rif^	0.5	<0.25	32	<0.5	0.06	<0.25	>1024^HLR^
*E.faecium* ST01109^Rif^	2	R	128	<0.5	0.5	<0.25	256
T1	R	R	R	2	R	R	64
T2	1	<0.25	R	R	0.13	2	64
T3	1	<0.25	R	R	0.5	<0.25	128
T4	R	R	R	R	R	R	128
ECOFF	VA	E	SM	TET	TMP	TE	KAN
*E.faecalis*	4	4	512	4	1	2	ND
*E.faecium*	4	4	128	4	ND	2	ND

*VA* vancomycin, *E* erythromycin, *SM* streptomycin, *TET* tetracycline, *TMP* trimethoprim, *TE* teicoplanin, *KAN* kanamycin. *ECOFF* Environmental cut-off [17]

^HLR,^ High level resistant*; ^R,^ Resistant

^Rif,^ Rifampicin resistant mutant; *ND* Not determined

Kanamycin resistance is ≥ 2000 μg/ml [35]

### Successful horizontal transfer of antibiotic multi-resistance amongst enterococci

Demonstration of phenotypic clumping ability successfully predicted the capture of horizontal gene transfer in three donor isolates. *E. faecalis* MW01105^Rif^ received horizontal gene transfer from the three donors MF06036, MF04010 and MF06035 (all *E. faecalis*). These reactions produced transconjugants T1, T2 and T4 respectively. Transconjugant T1 gained resistance to four of the six antibiotics (vancomycin, erythromycin, trimethoprim and teicoplanin) from its donor MF06036. Transconjugant T2 gained resistance to tetracycline from its donor MF04010. Transconjugant T4 gained resistance to the same four antibiotics (vancomycin, erythromycin, trimethoprim and teicoplanin) as its donor MF06035 (Table 4). Additionally, an interspecies transfer event (transconjugant T3) was captured with *E. faecium* ST01109^Rif^ receiving horizontal gene transfer from *E. faecalis* MF06036, gaining resistance to streptomycin and tetracycline only. Transfer efficiencies ranging from 10^−1^ to 10^−3^ were determined for transconjugants T1 and T4 and ranges of 10^−4^ to 10^−5^ for transconjugants T2 and T3. Several of the donor isolates readily reacted to the pheromone of all three recipient isolates (Table 3). However, despite MW02043^Rif^ inducing clumping in 42% of the donor isolates, isolation of transconjugants was unsuccessful. Transconjugant T1 was subjected to daily propagation on TSB for 12 weeks and as an endpoint it still retained its’ acquired vancomycin resistance (data not shown), demonstrating in one instance the preservation of acquired genes.

### PCR detection of resistance genes among transconjugants and their donors

Phenotypically, antibiotic resistances were efficiently transferred among environmental enterococcal isolates. To confirm these transfer events, PCR was carried out to identify these genes in the donors and their transconjugants (Table 5). As vancomycin resistance transfer was observed phenotypically, the *vanA* gene was tested for in the recipient MW01105^Rif^, its donors MF06036 and MF06035, and the transconjugants T1 and T4. The *vanA* gene was identified in the donors and the transconjugants but not in the recipient and there was a similar finding for the erythromycin resistance gene *ermB*. The recipients MW01105^Rif^ and ST01109^Rif^, the donors MF04010 and MF06036 and the transconjugants were all investigated for the presence of seven tetracycline genes (*tetK, tetL, tetM, tetO, tetS, tetT, tetW*). The tetracycline gene *tetL and tetM* were found in the donors with *tetM* being transferred to both T1 and T2 with only *tetL* being transferred to T2. None of these tetracycline genes were identified in the recipients.

**Table 5:** Transferred antibiotic resistance genes identified by PCR

Transconjugant	Donor	Recipient	Selection conditions (μg/ml)	Genes transferred	Efficiency
T1	MF06036	MW1105^Rif^	Vancomycin (10) + rifampicin (100)	vanA, ermB	7.8×10^−3^
T2	MF04010	MW1105^Rif^	Tetracycline (10) + rifampicin (100)	Tet M Tet L	2.3X10^−5^
T3	MF06036	ST01109^Rif^	Tetracycline (10) + rifampicin (100)	Tet M	1.8×10^−4^
T4	MF06035	MW1105^Rif^	Vancomycin (10) + rifampicin (100)	vanA. ermB	1.22×10^−1^

Solid plate mating reactions that resulted in the isolation of transconjugants. Donors and recipients were conjugated together on TSA and plated on selection media. Successful reactions were subjected to PCR and their transfer efficiency was calculated

## Discussion

We set out to establish if environmental enterococci possessed antimicrobial resistance genes and if they were able to pass them on to other naturally isolated enterococci. In pheromone dependant conjugation the phenotypical presentation of conjugation compatibility for an enterococcal plasmid donor is a characteristic cellular clumping reaction when exposed to a potential recipient. All enterococci were screened for clumping phenotypes and antibiotic resistance phenotypes.

The enterococci (Table 1) consisted of a combination of *E. faecalis* and *E. faecium* from varied sources in an agrarian system. Three pheromone producing isolates were selected based on their clumping of the selected donor isolates and their susceptible antimicrobial resistance profiles [22, 23]. In order to detect horizontal gene, transfer and capture transconjugants using antimicrobial selection, the recipient isolates required an antibiotic resistance that had universal susceptibility amongst the donor group. The group of potential donor isolates had both varied and prolific antimicrobial resistance phenotypes (Table 4) and Rifampicin resistance was chosen as it is caused by a point mutation rather than an acquired gene [24]. When comparing the donor isolates to ECOFF breakpoints (Table 4) it was clear that many of the resistance phenotypes were significantly higher than what is normally observed from the epidemiological data [17]. MF06035 and MF06036 both demonstrated high level resistance to several antibiotics including vancomycin (256 μg/ml and >512 μg/ml respectively) compared to lower ranges: 60–100 μg/ ml as reported by others [25]. Close to 50% of the isolates were resistant to at least two antimicrobials. No isolate was resistant to all of the antibiotics tested; however higher numbers of the isolates tested showed resistance to kanamycin and tetracycline and overall resistance at the higher concentrations were exhibited by isolates against erythromycin, streptomycin and kanamycin (Table 4). These findings agree with previous reports of a high frequency of antibiotic resistant enterococci in farm animals [26].

Successful conjugation reactions resulted in the isolation of four transconjugants (T1-T4). MIC’s carried out on the four transconjugants, highlighted all resistance phenotype transfers that occurred. Transconjugants 1 and 4 each had four resistance phenotypes transferred in one reaction which is rare as, at most, one or two resistance phenotype co-transfers have been reported [27]. With the growing concern that enterococci are a persistent nosocomial pathogen globally and their position as a highly competent reservoir for the transfer of serious virulence genes, this study demonstrates novel horizontal gene transfer events at high frequency.

The donor strain MF06036 was responsible for resistance transfer events resulting in different transconjugants (T1 and T3). Both conjugation reactions resulted in two different resistance transfer events and to our knowledge this is the first observation, of this kind, in enterococci isolated from an agrarian waterway ecosystem. This further demonstrates the broad range of transfer capability present within these enterococci. Transconjugant T1 retained its acquired vancomycin resistance, suggesting that pheromone responsive plasmids also code for maintenance and highly conserved replication systems [28] which supports their stability in transformed cells.

All transfer efficiencies in the generation of the transconjugants were within the ranges of the published literature [20, 29]. Transfer of vancomycin and/or erythromycin resistance was observed with a frequency of 10^−1^-10^−3^ obtained for donors isolated from poultry litter. In our hands the transfer frequency for tetracycline was only slightly lower (10^−4^-10^−5^). However, Vignaroli et al. [27] showed frequencies of 10^−6^-10^−9^ where, transfer occurred from donor strains isolated from animal faeces to a human *E. faecium* strain.

The resistant genes selected for in the current study (vancomycin, erythromycin and tetracycline) are coded on enterococcal mobile genetic elements and are of particular interest due to their association with nosocomial infections [12]. Transfer of antibiotic resistances from enterococci associated with food to those enterococci associated with the human gut has been reported [27] and *vanA* and *ermB* co-transfer from pig to human isolates has also been described [30]. In the current study MF06035, MF06036 and their transconjugants T1 and T4 tested positive for the *vanA* and *ermB* gene and MF04010, MF06036 and their transconjugants T2 and T3 carried the tetracycline genes *tetM, tetL* in line with the frequent location of these determinants on the same mobile genetic element, mainly transposons, in Gram positive bacteria [28]. To our knowledge this is the first report of the transfer of antibiotic resistances genes from farm associated *E. faecalis* to water catchment associated *E. faecium*.

## Conclusions

Within the environmental isolates examined, we see quite a diverse set of (potential) conjugation patterns. We have shown that multi-resistant organisms have greater conjugation potential than those isolates that exhibit a less resistant phenotype. The data suggested that this environment is a reservoir for strains that have the ability to conjugate and we have shown their ability to conjugate in vitro. Given the number of isolates investigated in this study, the potential for similar catchments to produce multiple antibiotic resistance strains is significant. In conclusion our findings show the importance of agrarian reservoirs of enterococci as sources of antibiotic resistance genes transmitted to human pathogens.

## Abbreviations

MIC: Minimum inhibitory concentration
TSA: Tryptone soy agar
TSB: Tryptone soya broth
